# Risk factors for mental disorders in pregnant women in two cities from São Paulo, Brazil: A cohort study

**DOI:** 10.1371/journal.pone.0330921

**Published:** 2025-09-04

**Authors:** Audêncio Victor, Maria Paula de Carvalho Leitão, Lívia Patrícia Rodrigues Batista, Laisla de França da Silva Teles, Perla Pizzi Argentato, Liania A. Luzia, Rinaldo Artes, Patrícia Helen Rondó

**Affiliations:** 1 Public Health Postgraduate Program, School of Public Health, University of São Paulo (USP), Avenida Doutor Arnaldo, 715, São Paulo, Brazil; 2 Nutrition Department, School of Public Health, University of São Paulo (USP), Avenida Doutor Arnaldo, 715, São Paulo, Brazil; 3 Insper - Institute of Education and Research, São Paulo, Brazil; 4 Faculty of Epidemiology and Population Health, London School of Hygiene and Tropical Medicine, London, United Kingdom; Mizan-Tepi University, ETHIOPIA

## Abstract

**Introduction:**

Mental disorders during pregnancy are a significant public health problem due to the substantial physiological and psychological changes that occur during this period. This study aims to investigate the risk factors for mental disorders in pregnant women by comparing data from two distinct cohorts in Jundiaí and Araraquara, Brazil.

**Methods:**

This is a prospective cohort study that included pregnant women from two Brazilian cohorts in São Paulo state. The Jundiaí cohort (1997–2000) included 865 pregnant women, while the Araraquara cohort (2017–2024) included 755 pregnant women. Socioeconomic, demographic, obstetric history, and mental health data were collected and analyzed. Mental health was assessed using standardized questionnaires, including the General Health Questionnaire (GHQ), the State-Trait Anxiety Inventory (STAI), Trait Anxiety Inventory (TAI) and the Perceived Stress Scale (PSS). Statistical analysis included bivariate tests and univariate and multivariate random-effects models for panel data.

**Results:**

Araraquara participants showed significantly higher GHQ scores at baseline (mean = 4.00) than Jundiaí (mean = 2.78; p < 0.001), with similar trends for SAI, TAI, and PSS. Scores decreased across visits in both cohorts (GHQ Visit 3: Coef. = –1.053, p < 0.001). Being single (GHQ: Coef. = 0.404, p = 0.019), separated/widowed (SAI: Coef. = 3.961, p = 0.005), lower education (TAI: Coef. = –1.910, p = 0.006), and higher household density (PSS: Coef. = 0.946, p = 0.012) were significant risk factors. Maternal morbidities such as urinary infections (TAI: Coef. = 0.862, p = 0.031), cervicitis/vaginitis (GHQ: Coef. = 0.290, p = 0.009), and tuberculosis (TAI: Coef. = 6.989, p = 0.033) were also strongly associated with worse mental health outcomes. Cohort differences remained significant even after adjustment (GHQ: Jundiaí vs Araraquara, Coef. = –1.357, p < 0.001).

**Conclusions:**

This study showed that pregnant women in the more recent Araraquara cohort exhibited significantly higher levels of psychological distress symptoms, anxiety, and perceived stress than those in the earlier Jundiaí cohort. These mental health outcomes were strongly associated with lower per capita income, lower education levels, higher household density, and adverse pregnancy conditions such as urinary infection and gestational hypertension. These findings highlight the worsening social vulnerability of pregnant women over time and reinforce the urgency of incorporating systematic mental health screening into prenatal care policies in Brazil.

## Introduction

Mental disorders during pregnancy, including symptoms of anxiety, depression, psychological distress, and perceived stress, are a growing concern in global public health [1,2], due to their effects on both maternal and child health outcomes [3–5]. Pregnancy is a particularly vulnerable period, marked by significant physiological, emotional, and social changes, which can increase the risk of developing or exacerbating mental health conditions [6–8]. In 2019, 970 million people, or one in eight, suffered from mental disorders, mainly anxiety and depressive disorders [9]. The burden is worse in low- and middle-income countries (LMICs), with approximately 80% of all people living with mental disorders in this region [10,11]. Nonetheless, mental disorders in LMICs are a neglected public health issue that significantly increases morbidity and mortality rates among mothers and newborns [11,12], particularly prevalent in women, especially during pregnancy; it ranges from 12 to 43% [9,13].

In Brazil, studies report that the prevalence of depressive symptoms during pregnancy ranges from 14% to 27% [14–17]. In comparison, anxiety symptoms affect between 19% and 40% of pregnant women [4,18]. These mental health conditions are particularly common among women of low socioeconomic status in urban centres such as São Paulo, with prevalence estimates for diagnosed mental disorders ranging from 17% to 23% [19]. A recent study conducted in São Paulo found that 78.1% of pregnant women receiving high-risk prenatal care exhibited elevated depressive symptoms, with protective factors including being in a stable relationship and having fewer previous pregnancies [20]. In the Brazilian context, intimate partner violence is a highly prevalent and chronic stressor that affects a substantial proportion of women during their reproductive years, has been consistently associated with the onset or worsening of mental disorders during pregnancy, particularly anxiety and depression [21,22].

Early identification of risk factors for these conditions is crucial for the implementation of effective interventions [8,23–25]. Prenatal depression, the most common mental disorder during pregnancy, can have significant adverse outcomes, including preterm birth, low birth weight, and obstetric complications [5,26]. Additionally, untreated depression during pregnancy can predispose the mother to develop postpartum depression, with prolonged impacts on the woman’s mental health and child development [27], Maternal stress and distress can affect gestational weight gain [14,15,28] and the child’s nutritional status [4].

Socioeconomic conditions play a pivotal role in shaping maternal mental health. Factors such as low income, limited educational attainment, high household density, racial inequalities, unemployment, and lack of social support have been consistently associated with mental disorders during pregnancy [7,8,23,25,26,29,30]. Conversely, a stable partnership, social protection, and safe housing function as protective factors [6,20]. These determinants, however, vary over time and across regions due to economic, cultural, and political shifts, underscoring the need for context-sensitive, longitudinal assessments [29,31].

Therefore, this study investigates risk factors for mental disorders in pregnant women using data from two cohorts, Jundiaí and Araraquara, to understand risk factors and trends for effective intervention. It employed validated screening tools to identify individuals at risk for mental disorders. While these instruments do not provide clinical diagnoses of depression or anxiety, they are widely used in epidemiological studies to identify individuals at risk for mental disorders during the perinatal period [6,25,26].

## Methods

### Study design

This is a prospective cohort study comparing two cohorts separated by a 20-year interval, respectively, in two municipalities with similar socioeconomic characteristics in the state of São Paulo, Brazil: Jundiaí Cohort Study (USP-MatStress): From an initial sample of 1182 women with gestational age ≤ 16 weeks who received prenatal care between September 1997 and August 2000 in 12 health units and five hospitals in the Municipality of Jundiaí, São Paulo, Brazil, 865 were followed quarterly in a cohort study until the birth of their children in one of the five hospitals in Jundiaí [4,5]. The Araraquara Cohort Study: The sample included women with a gestational age ≤ 19 weeks who received prenatal care in the 37 Basic Health Units and the Special Health Service (SESA) of the municipality of Araraquara, São Paulo, Brazil. The pregnant women participating in the mental health assessment, part of the Araraquara Cohort Study, were followed quarterly throughout prenatal care until the birth of their children from 05 January 2017 to 30 December 2024 [32].

The study conducted in Araraquara was approved by the Research Ethics Committee with Human Subjects at the School of Public Health, University of São Paulo, prior to data collection, under the protocol number CAEE: 59787216.2.0000.5421. The study conducted in Jundiaí was also approved 289/98, and informed written consent was obtained from all participants. The Ethical Committees of the School of Public Health, University of São Paulo, and the Health Secretariat of Jundiaí, SP approved the protocol.

## Outcome

### Mental health changes during pregnancy

Three standardized questionnaires were used to assess the mental health of pregnant women, measured at three visits during pregnancy (gestational age ≤ 16 or 19 weeks, 20–26, and 30–36 weeks). These included the general health questionnaire (GHQ), which screens for non-psychotic psychological distress [33,34], the State-Trait Anxiety Inventory (STAI), composed of the State Anxiety Inventory (SAI) and the Trait Anxiety Inventory (TAI), which assess both transient (state) and enduring (trait) anxiety [4,5], and the Perceived Stress Scale (PSS), which measures the individual’s perception of stress in daily life [35].

### Predictors

Several factors were considered as predictors for the study, including socioeconomic and demographic characteristics such as age, race (white, black, brown, yellow), marital status (single, separated/widowed, stable union), monthly family income in Brazilian minimum wages, per capita income, occupation, working status during pregnancy, the reason for not working (unemployed, maternity leave, others), the time spent working outside the home, and the hours worked both outside and inside the home. Additionally, the number of people per room and the level of education were considered.

Housing conditions included the type of house (owned, owned but not yet paid off, granted, other condition), the material of the house walls (brick), the presence of sewage, the number of rooms, and the possession of items such as a refrigerator, car, motorcycle, and access to piped water.

Obstetric history included the number of previous pregnancies, the time since the last delivery, the occurrence of previous abortions, previous stillbirths, and previous neonatal deaths. Pregnancy risk factors considered included morbidities such as hypertension, diabetes, rubella, urinary infection and pyelonephritis, syphilis, gonorrhea, cervicitis, vaginitis, tuberculosis, AIDS, and hepatitis. These variables were measured at three visits during pregnancy.

### Statistical analysis

Descriptive analyses included calculating means, standard deviations, medians, interquartile ranges, frequencies, and percentages of the studied variables distributed between the Araraquara and Jundiaí groups. Line graphs were used to visualize the evolution of mental changes over the visits. Due to strong asymmetry, the variables age, number of people per room, family income, and per capita income were included in the model on a logarithmic scale.

Models for unbalanced data with random effects in the panel were used [36]. as detailed below, including the effects of significant interactions between location and visit:


$$y_{ijt}=\alpha +\sum_{k=1}^{p}\beta_{k}\;x_{ijtk}+\gamma_{j}+\delta_{t}+{\gamma \delta }_{jt}+\varepsilon_{ijt}\;$$

where: $y_{ijt}\text{:}$ is the observed score for individual i, in location j (1 = Araraquara, 2 = Jundiaí), at visit t, t=1, 2, 3; $x_{ijtk}\text{:}$ is the observed value for covariate l k, for individual i, in location j e visita t; α: constant. $\beta_{k}$: is the parameter relative to the effect of covariate k;$\gamma_{j}\text{:}$ is the effect of location j ($\gamma_{1}=0);$ $\delta_{t}$: is the effect of visit t ($\delta_{1}=0);$ ${\gamma \delta }_{jt}\text{:}$ is the interaction effect between location j and visit t (${\gamma \delta }_{11},\;{\gamma \delta }_{12},\;{\gamma \delta }_{13},\;{\gamma \delta }_{21}$ e ${\gamma \delta }_{31}$ equal to zero); $\varepsilon_{ijt}\text{:}$ is the random error.

To accommodate the longitudinal design and repeated measures structure, we applied random-effects panel models (mixed models), allowing individual-specific intercepts and controlling for time-invariant unobserved heterogeneity. This approach suits unbalanced panels where not all individuals are observed in all visits. The dependent variables (GHQ, SAI, TAI, and PSS) were modelled as continuous outcomes to preserve variability and avoid loss of information that can result from categorization. This decision aligns with recommendations in epidemiological modelling. [36]. The model fit was performed following a step-by-step strategy, an iterative method that selects and removes independent variables, keeping those that presented a significance level of *p* < 0.02. All analyses were performed in Stata, version 18 (College Station, TX: Stata Corp LLC).

## Results

### Descriptive statistics of mental health scales

Table 1 presents the descriptive statistics of the mental health scales (GHQ, SAI, TAI, and PSS) across the three prenatal visits. At baseline (Visit 1), GHQ scores were higher in Araraquara (mean = 4.00) than in Jundiaí (mean = 2.78), indicating worse mental health status in the former. Similarly, SAI, TAI, and PSS scores differed between the cohorts. In both cities, scores decreased in subsequent visits. By Visit 2, GHQ scores dropped to 3.09 in Araraquara and 2.31 in Jundiaí. At Visit 3, this downward trend persisted, with GHQ scores reducing further to 2.89 in Araraquara and 2.46 in Jundiaí.

**Table 1:** Descriptive statistics of mental health assessment scales in the Jundiaí (1997-2000) and Araraquara (2017-2020) cohorts.

Visit	Statistics	Local							
		Jundiaí				Araraquara			
		GHQ	SAI	TAI	PSS	GHQ	SAI	TAI	PSS
1	Mean	2.78	37.47	42.28	23.07	4.00	41.75	44.01	23.59
	Standard Deviation	2.69	8.85	10.46	6.66	3.14	10.47	10.57	8.36
	Standard Error	0.09	0.30	0.36	0.28	0.11	0.38	0.38	0.30
	Median	2.00	36.00	41.00	23.00	3..00	40..00	43..00	23.00
	Minimum	0.00	19.00	8.00	5.00	0.00	20.00	21.00	2.00
	Maximum	12.00	77,00	78,00	44,00	12.00	77.00	79.00	50,00
	N	865	865	865	565	755	756	755	754
2	Mean	2.31	36.21	40.43	22.71	3.09	39.27	41.88	22.55
	Standard Deviation	2.60	8.32	10.16	6.97	2.90	9.67	10.71	8.22
	Standard Error	0.09	0.28	0.35	0.24	0.12	0.39	0.44	0.34
	Median	1.00	35.00	39.00	23.00	2.00	38.00	40.00	22.00
	Minimum	0.00	20.00	21.00	1.00	0.00	20.00	21.00	2.00
	Maximum	12.00	71.00	73.00	46.00	12.00	77.00	71.00	52.00
	N	865	865	865	864	599	601	600	600
3	Mean	2.46	37.13	39.80	22.27	2.89	39.36	40.28	21.59
	Standard Deviation	2.67	8.35	9.90	7.00	2.77	9.45	10.07	7.98
	Standard Error	0.09	0.28	0.34	0.24	0.12	0.40	0.43	0.34
	Median	2.00	36.00	39.00	22.00	2.00	38.00	38.00	21.00
	Minimum	0.00	20.00	3.00	1.00	0.00	20.00	20.00	2.00
	Maximum	12.00	69.00	68.00	53.00	12.00	71.00	80.00	52.00
	N	863	865	865	865	551	550	549	551

### Evolution of average mental health scores

**Fig 1:**
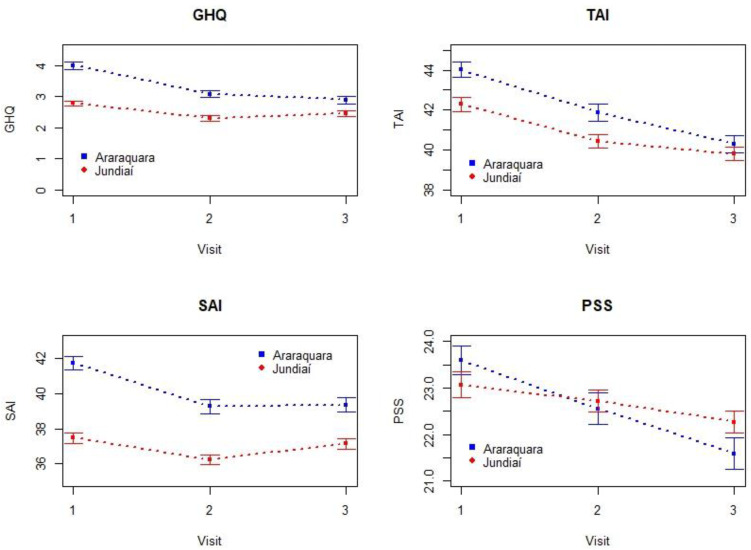
Profiles (mean and standard deviation) of the scales GHQ, SAI, TAI, and PSS over three visits in the Jundiaí and (1997-2000) and Araraquara (2017-2020) cohorts.

Fig 1. Displays the mean scores for each mental health instrument (GHQ, SAI, TAI, and PSS) across the three visits. Table 2 shows the results of model adjustments for location and visit effects. All scales showed significant decreases over time: scores were significantly lower in Visit 2 and Visit 3 compared to Visit 1 (p < 0.001 for all instruments). Additionally, scores were consistently lower in Jundiaí than in Araraquara (p < 0.05), suggesting better mental health in the former. For PSS, the location difference was only significant in Visit 3, where Jundiaí had higher scores than Araraquara (p < 0.05). Significant interaction effects between location and visit were observed, especially in Visit 3. These include GHQ (Coef. = 0.729), SAI (Coef. = 1.667), TAI (Coef. = 0.878), and PSS (Coef. = 1.940), all with p-values ≤ 0.001, indicating that the decline in scores over time was not uniform between cities.

**Table 2:** Parameter estimates of mental health assessment models in the Jundiaí (1997-2000) and Araraquara (2017-2020) cohorts.

Effect	GHQ	SAI	TAI	PSS
**Visit 2**	−0.878***	−2.227***	−2.005***	−1.035***
	[0.113]	[0.351]	[0.324]	[0.271]
**Visit 3**	−1.059***	−2.282***	−3.629***	−2.054***
	[0.116]	[0.362]	[0.335]	[0.280]
**Jundiaí**	−1.214***	−4.261***	−1.715***	−0,39
	[0.140]	[0.458]	[0.513]	[0.399]
**Visit 2 and Jundiaí**	0.400**	0.965*	0,153	0,578
	[0.148]	[0.461]	[0.424]	[0.382]
**Visit 3 and Jundiaí**	0.729***	1.940***	1.145**	1.154**
	[0.151]	[0.469]	[0.432]	[0.388]
**Constant**	3.996***	41.73***	44.00***	23.56***
	[0.102]	[0.334]	[0.374]	[0.273]
**N**	4498	4502	4499	4199

* P < 0.05; ** P < 0.01; *** P < 0.001. Standard error of the mean between brackets

### Factors associated with mental health changes

Table 3 presents the raw estimates from the mixed model, while Table 4 shows the adjusted effects considering all covariates. Several factors were associated with worse mental health. Being single was associated with higher GHQ (Coef. = 0.404, p = 0.019), SAI (Coef. = 1.623, p = 0.004), and TAI (Coef. = 1.427, p = 0.022) scores. Separated or widowed women also had higher scores across these scales, with notable effects in GHQ (Coef. = 1.120), SAI (Coef. = 3.961), and TAI (Coef. = 3.225). A higher number of people per room, measured on a logarithmic scale, was positively associated with GHQ (Coef. = 0.281, p = 0.042), SAI (Coef. = 1.138, p = 0.013), and PSS (Coef. = 0.946, p = 0.012) scores. Higher education (high school or more) was associated with lower anxiety scores: SAI (Coef. = −1.416, p = 0.016) and TAI (Coef. = −1.910, p = 0.006).

**Table 3:** Parameter estimates of model (1) for factors associated with mental health changes (marginal models).

Variables	GHQ				SAI				TAI					PSS			
	Coef	Std.err	z	P	Coef	Std.err	z	P	Coef	Std.err	z	P	Coef		Std.err	z	P
Pre-gestational weight	−0.006	0.004	−1.35	0.177	−0.026	0.015	−1.76	0.079	−0.056	0.017	−3.24	0.001	−0.031		0.012	−2.51	0.012
Height	−0.021	0.014	−1.5	0.133	−0.007	0.047	−0.15	0.882	−0.068	0.055	−1.25	0.213	0.007		0.039	0.17	0.862
Pre-gestational BMI	−0.013	0.012	−1.05	0.294	−0.076	0.040	−1.88	0.06	−0.127	0.048	−2.66	0.008	−0.075		0.034	−2.24	0.025
Current weight	−0.005	0.004	−1.25	0.212	−0.018	0.014	−1.24	0.214	−0.045	0.017	−2.69	0.007	−0.028		0.012	−2.3	0.021
Current BMI	−0.007	0.011	−0.65	0.514	−0.041	0.038	−1.09	0.278	−0.082	0.042	−1.95	0.052	−0.052		0.031	−1.67	0.095
BMI variation	0.546	0.536	1.02	0.309	2.935	1.709	1.72	0.086	1.798	1.667	1.08	0.281	1.254		1.365	0.92	0.358
Age	0.049	0.238	0.21	0.837	0.272	0.800	0.34	0.734	−2.375	0.950	−2.5	0.012	−1.779		0.671	−2.65	0.008
Race (ref. White)																	
Black	0.246	0.227	1.08	0.278	−0.213	0.759	−0.28	0.779	−0.897	0.900	−1	0.319	−0.396		0.637	0.62	0.534
Brown	0.206	0.143	1.45	0.147	1.031	0.478	2.16	0.031	1.589	0.567	2.8	0.005	0.240		0.401	0.6	0.549
Yellow	−0.141	0.849	−0.17	0.868	0.677	2.841	0.24	0.812	−4.541	3.351	−1.35	0.175	−2.537		2.376	−1.07	0.286
Marital Status (ref. Married)																	
Single	0.424	0.158	2.67	0.007	1.649	0.525	3.14	0.002	1.786	0.590	3.03	0.002	1.029		0.435	2.36	0.018
Separated/Widowed	1.443	0.421	3.43	0.001	4.758	1.370	3.47	0.001	4.059	1.547	2.62	0.009	2.486		1.151	2.16	0.031
Stable union	0.419	0.120	3.49	0.000	1.174	0.396	2.96	0.003	1.124	0.436	2.58	0.010	0.884		0.326	2.71	0.007
Income	−0.303	0.084	−3.6	0.000	−0.859	0.279	−3.08	0.002	−1.405	0.310	−4.53	0.000	−0.977		0.229	−4.26	0.000
Per Capita Income	−0.442	0.075	−5.87	0.000	−1.292	0.251	−5.15	0	−1.641	0.281	−5.84	0.000	−1.206		0.207	−5.84	0.000
Working during pregnancy	−0.202	0.100	−2.03	0.043	−0.209	0.322	−0.65	0.517	−0.713	0.332	−2.15	0.032	−0.836		0.267	−3.13	0.002
Work (ref. Working)																	
Unemployed	0.194	0.117	1.66	0.096	0.462	0.377	1.22	0.221	0.680	0.389	1.75	0.080	0.925		0.312	2.96	0.003
Maternity leave	0.934	0.367	2.54	0.011	0.494	1.139	0.43	0.664	2.059	1.076	1.91	0.056	1.664		0.904	1.84	0.066
Others	0.153	0.116	1.32	0.186	−0.052	0.374	−0.14	0.889	0.567	0.382	1.48	0.138	0.598		0.309	1.93	0.053
Working hours	0.001	0.003	0.53	0.595	0.004	0.009	0.47	0.638	0.007	0.009	0.81	0.420	0.010		0.007	1.48	0.138
People per room	0.620	0.121	5.13	0.000	2.145	0.400	5.36	0	2.055	0.450	4.57	0.000	1.793		0.331	5.41	0.000
High school or more	−0.363	0.167	−2.17	0.030	−2.269	0.559	−4.06	0	−3.365	0.661	−5.09	0.000	−1.833		0.469	−3.91	0.000
House ownership																	
Paid owned house	−0.204	0.128	−1.59	0.112	−0.592	0.423	−1.4	0.162	−0.670	0.469	−1.43	0.153	−0.183		0.349	−0.52	0.600
Unpaid owned house	0.246	0.175	−1.41	0.158	−0.243	0.572	−0.43	0.671	−0.653	0.621	−1.05	0.293	−0.955		0.466	−2.05	0.041
Granted house	0.096	0.178	0.54	0.587	0.156	0.586	0.27	0.79	−0.454	0.644	−0.7	0.481	0.281		0.481	0.58	0.559
Occupied house or other condition	0.250	0.419	0.6	0.550	0.692	1.323	0.52	0.601	0.494	1.342	0.37	0.713	1.073		1.069	1	0.316
Brick house	−1.220	0.354	−3.45	0.001	−2.706	1.144	−2.37	0.018	−3.135	1.227	−2.55	0.011	−2.087		0.946	−2.21	0.027
Sewage system	−0.173	0.202	−0.86	0.392	−0.025	0.671	−0.04	0.971	−0.259	0.767	−0.34	0.736	−0.272		0.561	−0.49	0.628
Number of rooms	−0.026	0.034	−0.77	0.440	−0.227	0.113	−2.01	0.044	−0.232	0.124	−1.87	0.062	−0.246		0.093	−2.65	0.008
Refrigerator ownership	−0.629	0.296	−2.13	0.033	−2.632	0.978	−2.69	0.007	−3.236	1.090	−2.97	0.003	−2.719		0.814	−3.34	0.001
Car ownership	−0.449	0.111	−4.05	0.000	−0.921	0.368	−2.5	0.012	−1.787	0.412	−4.34	0.000	−1.155		0.304	−3.8	0.000
Motorcycle ownership	−0.265	0.456	−0.58	0.561	−1.350	1.533	−0.88	0.379	−2.689	1.837	−1.46	0.143	−1.517		1.303	−1.16	0.244
Tap water inside the house	−0.183	0.228	−0.800	0.422	0.334	0.767	0.430	0.664	0.620	0.917	0.680	0.499	−0.540		0.652	−0.830	0.408
Number of pregnancies	0.266	0.044	6.04	0.000	0.912	0.148	6.16	0	0.822	0.177	4.63	0.000	0.522		0.125	4.17	0.000
Previous pregnancies	0.451	0.116	3.88	0.000	1.399	0.390	3.59	0	1.057	0.466	2.27	0.023	0.893		0.328	2.72	0.006
Time since last delivery																	
Less than 1 year	0.284	0.521	0.54	0.586	1.390	1.799	0.77	0.44	0.639	1.987	0.32	0.748	−0.345		1.485	−0.23	0.816
1 to 1.5 years	0.774	0.603	1.28	0.199	4.054	2.091	1.94	0.052	3.248	2.326	1.4	0.163	2.254		1.732	1.3	0.193
1.5 to 2 years	0.037	0.623	0.06	0.952	0.766	2.157	0.36	0.722	−1.962	2.396	−0.82	0.413	−0.462		1.786	−0.26	0.796
More than 2 years	0.488	0.197	2.48	0.013	1.754	0.680	2.58	0.01	1.064	0.754	1.41	0.159	0.371		0.563	0.66	0.511
Abortion (ref. Never pregnant)																	
No	0.360	0.127	2.84	0.004	0.983	0.425	2.31	0.021	0.706	0.508	1.39	0.165	0.695		0.358	1.94	0.052
Yes	0.619	0.163	3.8	0.000	2.281	0.547	4.17	0	1.763	0.654	2.7	0.007	1.310		0.460	2.85	0.004
Stillbirth (ref. Never pregnant)																	
No previous stillborn	0.450	0.117	3.85	0.000	1.424	0.392	3.63	0	1.078	0.469	2.3	0.022	0.897		0.330	2.72	0.007
Previous stillborn	0.345	0.496	0.7	0.486	−0.206	1.665	−0.12	0.902	−0.207	1.983	−0.1	0.917	0.828		1.391	0.6	0.551
Neonatal death (ref. Never pregnant)																	
No previous neonatal death	0.448	0.117	3.84	0.000	1.376	0.392	3.51	0	1.094	0.468	2.34	0.019	0.932		0.330	2.83	0.005
Previous neonatal death	0.247	0.540	0.46	0.647	1.685	1.816	0.93	0.353	−1.642	2.166	−0.76	0.448	−1.127		1.531	−0.74	0.462
Low Birth Weight-LBW (ref. Never pregnant)																	
No previous LBW	0.437	0.117	3.72	0.000	1.280	0.394	3.25	0.001	0.968	0.472	2.05	0.040	0.924		0.332	2.78	0.005
Previous LBW	0.584	0.346	1.69	0.091	3.282	1.167	2.81	0.005	2.338	1.393	1.68	0.093	0.254		0.985	0.26	0.796
Preterm babies (ref. Never pregnant)																	
No previous preterm babies	0.431	0.118	3.64	0.000	1.358	0.397	3.42	0.001	0.932	0.475	1.96	0.050	0.902		0.335	2.7	0.007
Previous preterm babies	0.600	0.282	2.13	0.033	1.665	0.949	1.75	0.079	2.246	1.128	1.99	0.046	0.764		0.795	0.96	0.336
Hypertension in pregnancy	0.421	0.177	2.38	0.017	0.968	0.565	1.71	0.086	0.661	0.557	1.19	0.235	0.672		0.465	1.45	0.148
Diabetes in pregnancy	0.129	0.292	0.44	0.660	0.309	0.942	0.33	0.743	−0.198	0.941	−0.21	0.834	0.648		0.747	0.87	0.386
Rubella in pregnancy	0.908	1.153	0.79	0.431	1.588	3.617	0.44	0.661	2.436	3.375	0.72	0.470	4.503		2.803	1.61	0.108
Urinary infection and pyelonephritis in pregnancy	0.359	0.132	2.72	0.007	0.546	0.416	1.31	0.19	0.964	0.396	2.43	0.015	1.051		0.336	3.13	0.002
Syphilis in pregnancy	0.034	0.455	0.07	0.941	2.369	1.449	1.63	0.102	−0.850	1.417	−0.6	0.549	0.850		1.142	0.75	0.456
Gonorrhea in pregnancy	−0.711	1.332	−0.53	0.593	4.418	4.173	1.06	0.29	−0.903	3.894	−0.23	0.817	1.531		3.238	0.47	0.636
Cervicitis/vaginitis in pregnancy	0.293	0.110	2.67	0.008	0.815	0.347	2.35	0.019	0.974	0.332	2.93	0.003	0.423		0.290	1.46	0.144
Tuberculosis in pregnancy	3.001	0.997	3.01	0.003	7.051	3.142	2.24	0.025	7.649	2.961	2.58	0.010	2.578		3.175	0.81	0.417
HIV/AIDS in pregnancy	1.007	1.028	0.98	0.327	5.607	3.394	1.65	0.099	−0.942	3.777	−0.25	0.803	0.930		2.778	0.33	0.738
Hepatitis in pregnancy	−1.217	0.950	−1.28	0.200	−2.650	3.016	−0.88	0.38	−4.288	2.914	−1.47	0.141	−2.429		2.397	−1.01	0,311

**Table 4:** Multivariable panel data models for factors associated with mental health changes in the Jundiaí (1997–2000) and Araraquara (2017–2020) Cohorts.

Variable	GHQ			SAI			TAI			PSS		
	Coef.	P	Conf. Interval	Coef.	P	Conf. Interval	Coef.	P	Conf. Interval	Coef.	P	Conf. Interval
**Pre-gestaional weight**	−0.0111	0.365	[-0.0352,0.0130]	−0.0897*	0.025	[-0.168,-0.0115]	−0.0938*	0.023	[-0.175,-0.0128]	−0.042	0.198	[-0.106,0.0220]
**Current weight**	0.00532	0.661	[-0.0185,0.0291]	0.0671+	0.087	[-0.00965,0.144]	0.0529	0.182	[-0.0247,0.130]	0.0199	0.534	[-0.0429,0.0827]
**Age**	−0.15	0.635	[-0.771,0.471]	0.0414	0.969	[-2.033,2.116]	−2.652*	0.031	[-5.068,-0.236]	−2.066*	0.020	[-3.803,-0.329]
**Race (ref. White)**												
Black	0.174	0.441	[-0.269,0.617]	−0.433	0.567	[-1.916,1.049]	−1.137	0.201	[-2.882,0.607]	−0.611	0.336	[-1.854,0.632]
Brown	0.0908	0.526	[-0.190,0.371]	0.601	0.210	[-0.338,1.541]	1.068+	0.058	[-0.0377,2.174]	−0.14	0.728	[-0.927,0.648]
Yellow	−0.067	0.936	[-1.707,1.573]	0.768	0.784	[-4.716,6.252]	−3.988	0.224	[-10.42,2.443]	−2.187	0.350	[-6.775,2.401]
**Marital status (ref. Married)**												
Single	0.404*	0.019	[0.0674,0.741]	1.623**	0.004	[0.512,2.735]	1.427*	0.022	[0.203,2.651]	0.717	0.125	[-0.199,1.634]
Separated/Widowed	1.120**	0.008	[0.290,1.950]	3.961**	0.005	[1.220,6.702]	3.225*	0.036	[0.205,6.245]	1.832	0.113	[-0.432,4.096]
Stable Union	0.289*	0.022	[0.0410,0.537]	0.755+	0.070	[-0.0612,1.571]	0.449	0.322	[-0.440,1.337]	0.337	0.324	[-0.333,1.008]
**High School or more**	0.0299	0.865	[-0.316,0.376]	−1.416*	0.016	[-2.573,-0.260]	−1.910**	0.006	[-3.265,-0.554]	−0.755	0.127	[-1.725,0.215]
**Working during Pregnancy (ref. Working)**												
Unemployed	0.0484	0.688	[-0.188,0.284]	0.0396	0.919	[-0.725,0.804]	0.148	0.711	[-0.633,0.928]	0.546+	0.091	[-0.0875,1.179]
Maternity Leave	0.865*	0.018	[0.148,1.581]	0.393	0.732	[-1.862,2.649]	2.011+	0.059	[-0.0791,4.102]	1.554+	0.087	[-0.223,3.331]
Others	0.00459	0.970	[-0.233,0.242]	−0.454	0.247	[-1.221,0.314]	0.132	0.740	[-0.645,0.908]	0.189	0.560	[-0.446,0.823]
**People per Room**	0.281*	0.042	[0.00958,0.552]	1.138*	0.013	[0.244,2.031]	0.717	0.150	[-0.259,1.693]	0.946*	0.012	[0.210,1.683]
**Brick House**	−0.857*	0.017	[-1.563,-0.150]	−1.7	0.148	[-4.004,0.605]	−1.865	0.130	[-4.280,0.551]	−1.255	0.194	[-3.149,0.638]
**Refrigerator Ownership**	−0.253	0.405	[-0.850,0.344]	−1.746+	0.082	[-3.716,0.224]	−1.762	0.110	[-3.926,0.402]	−1.699*	0.042	[-3.338,-0.0604]
**Car Ownership**	−0.263*	0.023	[-0.490,-0.0355]	−0.238	0.534	[-0.990,0.513]	−0.940*	0.027	[-1.771,-0.109]	−0.568+	0.073	[-1.189,0.0526]
**Number of previous prenancies**	0.235**	0.002	[0.0894,0.380]	0.754**	0.002	[0.268,1.240]	0.985***	0.001	[0.417,1.554]	0.453*	0.029	[0.0451,0.860]
**AbortionAborto (ref. Never Pregnant)**												
No	0.0425	0.803	[-0.292,0.377]	−0.254	0.657	[-1.375,0.866]	−0.252	0.709	[-1.571,1.068]	0.362	0.450	[-0.578,1.303]
Yes	0.117	0.618	[-0.342,0.575]	0.439	0.575	[-1.094,1.973]	0.00509	0.996	[-1.802,1.813]	0.692	0.292	[-0.595,1.979]
**Diabetes in pregnancy**	0.115	0.697	[-0.465,0.696]	0.602	0.528	[-1.269,2.473]	0.0779	0.934	[-1.770,1.926]	0.783	0.301	[-0.702,2.269]
**Hypertension in pregnancy**	0.393*	0.032	[0.0343,0.753]	0.658	0.262	[-0.491,1.807]	0.535	0.351	[-0.589,1.660]	0.568	0.241	[-0.381,1.516]
**Urinary infection and pyelonephrytis in pregnancy**	0.286*	0.032	[0.0245,0.548]	0.448	0.289	[-0.380,1.275]	0.862*	0.031	[0.0778,1.645]	0.931**	0.006	[0.264,1.598]
**Cervicitis, vaginitis in pregnancy**	0.290**	0.009	[0.0718,0.509]	0.677+	0.056	[-0.0165,1.370]	0.754*	0.025	[0.0944,1.414]	0.232	0.431	[-0.346,0.811]
**Tuberculosis in pregnancy**	2.837*	0.010	[0.671,5.003]	6.739+	0.054	[-0.108,13.59]	6.989*	0.033	[0.562,13.42]	1.108	0.729	[-5.157,7.372]
**Visit (ref. Visit 1**)												
Visit 2	−0.875***	0.000	[-1.118,-0.631]	−2.265***	0.000	[-3.030,-1.500]	−2.045***	0.000	[-2.759,-1.332]	−1.014***	0.001	[-1.612,-0.415]
Visit 3	−1.053***	0.000	[-1.359,-0.746]	−2.607***	0.000	[-3.580,-1.634]	−3.825***	0.000	[-4.759,-2.890]	−1.959***	0.000	[-2.732,-1.187]
**Local (ref. Araraquara)**												
Jundiaí	−1.357***	0.000	[-1.698,-1.016]	−4.466***	0.000	[-5.587,-3.346]	−2.540***	0.000	[-3.785,-1.295]	−0.983*	0.045	[-1.942,-0.0234]
Interaction EffectsEfeitos de interação (Visit* Local)												
Visit 2 – Jundiaí	0.372*	0.014	[0.0753,0.669]	0.735	0.120	[-0.193,1.663]	0.0265	0.951	[-0.820,0.873]	0.321	0.412	[-0.446,1.088]
Visit 3 – Jundiaí	0.646***	0.000	[0.340,0.952]	1.667***	0.001	[0.710,2.624]	0.878*	0.049	[0.00193,1.754]	0.636	0.114	[-0.152,1.424]
Constant	5.583***	0.000	[3.427,7.739]	45.43***	0.000	[38.26,52.60]	58.03***	0.000	[49.82,66.24]	34.16***	0.000	[28.18,40.14]
**N**	4336			4339			4337			4070		

Note: The variables age, number of people per room, income, and income per capita were included in the model on a logarithmic scale. p < 0.05*, p < 0.01**, p < 0.001***. Ref = reference. Coef. = Coefficient, P = p-value, and Conf. Interval = Confidence Interval.

Pre-gestational weight was associated with better mental health, particularly lower SAI (Coef. = −0.0897, p = 0.025) and TAI (Coef. = −0.0938, p = 0.023) scores, while current weight showed a borderline positive association with anxiety (Coef. = 0.0671, p = 0.087). Age, also log-transformed, was associated with lower scores in TAI (Coef. = −2.652, p = 0.031) and PSS (Coef. = −2.066, p = 0.020). Obstetric history also mattered: the number of previous pregnancies was positively associated with higher scores in GHQ (Coef. = 0.235, p = 0.002), SAI (Coef. = 0.754, p = 0.002), TAI (Coef. = 0.985, p < 0.001), and PSS (Coef. = 0.453, p = 0.029).

Regarding maternal morbidities, hypertension was linked to worse GHQ scores (Coef. = 0.393, p = 0.032). Urinary infections and pyelonephritis were associated with increased scores in GHQ (Coef. = 0.286), TAI (Coef. = 0.862), and PSS (Coef. = 0.931). Cervicitis and vaginitis during pregnancy were linked to higher GHQ (Coef. = 0.290), SAI (Coef. = 0.677), and TAI (Coef. = 0.754) scores. Tuberculosis showed a strong association with increased GHQ (Coef. = 2.837, p = 0.010) and TAI (Coef. = 6.989, p = 0.033) scores.

After adjusting for all covariates, mental health scores remained significantly lower in Visits 2 and 3 across all instruments compared to Visit 1. For GHQ, the coefficients were −0.875 (p < 0.001) in Visit 2 and −1.053 (p < 0.001) in Visit 3. For SAI, the coefficients were −2.265 and −2.607, both with p < 0.001. For TAI, the reductions were −2.045 and −3.825, and for PSS, −1.014 and −1.959, all with p < 0.001. Jundiaí continued to show significantly lower scores compared to Araraquara in GHQ (Coef. = −1.357, p < 0.001), SAI (Coef. = −4.466, p < 0.001), TAI (Coef. = −2.540, p < 0.001), and PSS (Coef. = −0.983, p = 0.045).

## Discussion

This study, based on two Brazilian cohorts 20 years apart, found that pregnant women from Araraquara had significantly worse mental health scores than those from Jundiaí. Poor mental health was associated with being single, low education, crowded housing, and obstetric morbidities such as hypertension, infections, and tuberculosis

Mental health during pregnancy has become an increasing global concern. The World Health Organization (WHO) emphasizes the importance of addressing maternal mental health due to its significant impacts on maternal and neonatal outcomes [37]. Mental disorders such as psychological distress symptoms, anxiety, and perceived stress are prevalent during pregnancy and have been associated with adverse outcomes for both mother and baby [4,26,31,38,39]. Studies such as that by REDINGER et al, also highlight the high prevalence of symptoms of depression and anxiety in the first trimester of pregnancy, corroborating our findings [29]. Higher pre-gestational weight was associated with lower anxiety scores, while a higher number of previous pregnancies correlated with higher scores on all mental health measures. The relationship between nutritional status and maternal mental health is complex and multifaceted, requiring an integrated approach to understand these mechanisms [18,28,39,40]. The study conducted by PASKULIN et al (2017), observed associations between dietary patterns and mental disorders in pregnant women, emphasizing the importance of adequate nutrition [18]. Several contextual and temporal factors may explain these differences.

### Socioeconomic conditions and austerity

Socioeconomic factors such as lower income, lower educational level, and higher household density were significantly associated with higher levels of psychological distress symptoms, anxiety, and perceived stress in pregnant women from Araraquara. These results are consistent with previous studies that identified low income and lack of social support as critical predictors of mental disorders during pregnancy [7,8,23,25,30,37,41]. The presence of a partner and a robust social network are important protective factors against these disorders. FARIAS et al (2021) also observed that maternal mental health is closely linked to socioeconomic conditions, corroborating our findings [14]. Brazil has changed over the past twenty years and may have increased the vulnerability of low-income pregnant women. Rising inequality, austerity, and limited social policies have created structural barriers to mental well-being [16,42]. Second, the effects of accelerated urbanisation, such as overcrowded housing, reduced green spaces, and fragmented social networks, may increase the risk of common mental disorders [42,41]. Third, the underdevelopment of early mental health screening programs and psychosocial support within prenatal services may have led to delayed diagnoses and care [43,44].

### Urbanization, isolation, and loneliness

Urbanisation and globalization, driven by liberal economic policies, have transformed Brazilian metropolises in recent decades, resulting in significant growth. However, these changes have also led to an increase in loneliness and social isolation, which are critical factors profoundly affecting mental health. Recent studies have highlighted that rapid urbanization and the social changes resulting from globalization contribute to loneliness and stress, exacerbating mental health problems in large cities [42]. Loneliness is a significant risk factor for various mental illnesses, including symptoms of depression and anxiety, and is exacerbated by the urban environment, which often promotes isolation [45].

### Climate change, COVID-19 pandemic, and perinatal morbidity

Health complications during pregnancy, such as urinary infection, pyelonephritis, cervicitis, vaginitis, tuberculosis, and hypertension, were associated with worse mental health scores. These results are in line with existing literature showing that health complications can exacerbate stress and anxiety during pregnancy [8,20,26,41]. Moreover, gestational hypertension was associated with higher GHQ scores, highlighting the interaction between physical and mental health. GOMES et al (2023), also reported that pregnant women with health complications are more likely to develop mental disorders [15]. The COVID-19 pandemic exacerbated mental health problems globally, with increased stress, insomnia, anxiety, and depression. The COVID-19 pandemic further exacerbated mental distress, and emerging evidence suggests its psychological effects may persist in the post-pandemic era [46]. Additionally, i recent years, the planet has experienced climate changes that have significant effects on mental health. Extreme climatic events such as floods and storms, and chronic stresses such as extreme heat and drought affect mental health, leading to anxiety disorders, depression, and post-traumatic stress disorder [47].

The findings of this study underscore that the integration of validated instruments GHQ, STAI, and PSS facilitates a multidimensional assessment of psychological well-being. While each instrument evaluates specific domains, they have been shown to complement one another in perinatal mental health research, providing a more robust picture of emotional distress [48,49], and the urgent need for targeted, evidence-based interventions aimed at pregnant women in vulnerable conditions. Public policies should prioritise economic security, adequate housing, and equitable access to quality education as foundational determinants of maternal mental health. Moreover, it is imperative to institutionalize the integration of mental health screening and care into routine prenatal services, ensuring early identification and timely treatment of psychological distress and comorbid conditions. Building upon international experiences, Kinser et al (2018) highlight the effectiveness of structured screening tools and intervention protocols for perinatal distress, stress, and anxiety symptoms which could be adapted and scaled within the Brazilian Unified Health System. Such measures not only improve maternal well-being but also enhance fetal and neonatal health outcomes, reinforcing the need for a comprehensive, intersectoral response to maternal mental health [27].

This study has some limitations. The analysis was based on data from two specific cohorts, which may limit the generalizability of the findings to other populations. Additionally, unmeasured variables, such as specific forms of social support and exposure to stressful life events, may have influenced the outcomes. Another important methodological limitation is the use of self-reported screening tools (GHQ, STAI, and PSS) to assess mental health symptoms. These instruments are designed to detect nonspecific psychological distress and do not replace clinical diagnosis or psychiatric evaluation. Therefore, the results should be interpreted with caution regarding the identification of mental disorders. Future studies should consider incorporating validated diagnostic assessments, as well as additional psychosocial and environmental variables, to enable a more comprehensive understanding of mental health during pregnancy. Existing literature, including the work of LANCASTER et al. (2010), emphasizes that a more holistic approach can provide deeper insights into the complexity of maternal mental health [25].

## Conclusion

This study showed that pregnant women in Araraquara experienced significantly higher levels of psychological distress symptoms, anxiety, and perceived stress. This may reflect changes in Brazil’s socioeconomic landscape and disparities between cohorts. Over the last 20 years, increased economic inequality, precarious employment, urban overcrowding, and weakened social support networks have likely worsened stressors for pregnant women today. Lower income, lower education, higher household density, and pregnancy-related morbidities correlated with poorer mental health. In contrast, higher pre-gestational weight is linked to lower anxiety, and more prior pregnancies are associated with increased distress across assessment scales. These findings highlight the necessity for context-sensitive public policies. Enhancing economic stability, housing quality, and educational access, along with integrating mental health services into prenatal care, is crucial for addressing maternal mental health burdens. By tackling both structural and clinical determinants, these interventions can reduce risks and foster maternal and neonatal well-being over time and across contexts.

## Supporting information

S1 Strobe Project administration: Patrícia Helen Rondó. (DOCX)

S1 Checklist Resources: Patrícia Helen Rondó. (DOCX)
