# Supplementary material for: Risk factors for mental disorders in pregnant women in two cities from São Paulo, Brazil: A cohort study
Source: PLoS One. 2025 Sep 4;20(9):e0330921. doi: 10.1371/journal.pone.0330921 (PMC12410748; doi:10.1371/journal.pone.0330921)
Supplement: S1 Checklist — (DOCX) [file pone.0330921.s002.docx]

**Human Participants Research Checklist**

***Complete the following if your study involved human participants or human participants’ data. These questions should be addressed for prospective and retrospective studies.***

1. Did you obtain ethics approval for this study?

Yes. The study was approved by the Research Ethics Committee with Human Subjects at the School of Public Health, University of São Paulo, under protocol number CAEE: 59787216.2.0000.5421, opinion number 1.885.874. Issue Date: 05 January 2017

Uploaded ___ N/A

- - If you did not obtain ethical approval, please explain why this was not required below.

1. If you prospectively recruited human participants for the study – for example, you conducted a clinical trial, distributed questionnaires, or obtained tissues, data or samples for the purposes of this study, please report in the Methods:

i. Recruitment period:

The recruitment period for the study began in January 2017 and ended in December 2022.

ii. Participant consent:

Participants provided informed written consent (ICF). The consent was documented by signature, and the participants were informed of the study's purpose, risks, and benefits. Consent for minors was obtained from parents or guardians. No waiver for consent was required by the ethics committee

X Completed ___ N/A

1. If you are reporting a retrospective study of medical records or archived samples, please report in the Methods section:

i. Date of data access:

The data were accessed for research purposes in January 2024.

ii. Participant anonymity:

All data were fully anonymized before the researchers accessed it, ensuring no individual participant could be identified during or after the data collection.

X Completed ___ N/A
